# Calcium Phosphate Mineralization of Bulk Alginate Hydrogels Composites With Metal and Metal Oxide Nanoparticles

**DOI:** 10.1002/mabi.202500468

**Published:** 2026-01-20

**Authors:** Ana‐Marija Milisav, Vida Strasser, Andrea Marfoglia, Krunoslav Bojanić, Ina Erceg, Silke Christiansen, Maja Ivanić, Željka Fiket, Sophie Cazalbou, Maja Dutour Sikirić

**Affiliations:** ^1^ Division of Physical Chemistry, Ruđer Bošković Institute Bijenička c. 54 Zagreb Croatia; ^2^ CIRIMAT and Laboratoire de Génie Chimique Université de Toulouse CNRS, Toulouse INP 31062 Cedex 09 Toulouse France; ^3^ Department of Medical Surgical and Health Sciences University of Trieste 34139 Trieste Italy; ^4^ International Centre for Genetic Engineering and Biotechnology (ICGEB) 34149 Trieste Italy; ^5^ Division of Materials Chemistry, Ruđer Bošković Institute Bijenička c. 54 Zagreb Croatia; ^6^ Fraunhofer Institute For Ceramic Technologies and Systems IKTS Äußere Nürnberger Strasse 62 Forchheim Germany; ^7^ Innovations‐Institut für Nanotechnologie Und Korrelative Mikroskopie Äußere Nürnberger Strasse 62 Forchheim Germany; ^8^ Division for Marine and Environmental Research Ruđer Bošković Institute Bijenička c. 54 Zagreb Croatia

**Keywords:** alginate hydrogels, antibacterial activity, calcium phosphates, metal nanoparticles

## Abstract

Alginate hydrogels are promising materials for biomedical applications due to their biocompatibility and ability to mimic the extracellular matrix. However, poor mechanical stability and limited bioactivity hinder their wider clinical application. This problem can be overcome by incorporating nanoparticles (NPs) and calcium phosphates (CaPs). In this study, the simultaneous gelation and CaP mineralization of bulk alginate hydrogels in the presence of antimicrobial silver (AgNP), copper oxide (CuONP), and zinc oxide (ZnONP) was investigated. Calcium‐deficient hydroxyapatite forms at pH 7.4, and stable amorphous calcium phosphate at pH 9.0. The incorporation of NPs influences the morphology of mineral phases but not composition. Rheological testing revealed that mineralized hydrogels exhibit earlier network breakdown compared to the non‐mineralized ones, with critical strain values dependent on both pH and NP type. Ion release is pH‐dependent, with generally higher metal ion release from non‐mineralized hydrogels. Antibacterial assays demonstrate significant inhibition of *S. aureus* by hydrogels prepared at pH 9.0, except for hydrogels containing CuONPs. For *P. aeruginosa*, the differences in inhibition rates between different hydrogels were less pronounced. The obtained results indicate that CaP‐mineralized alginate hydrogels incorporating metal and metal oxide NPs exhibit tunable properties, confirming their potential for bone tissue engineering applications and infection prevention.

## Graphical abstract

This study explores alginate hydrogels simultaneously mineralized with calcium phosphates and embedded with antimicrobial nanoparticles (AgNPs, CuONPs, ZnONPs). The materials are synthesized at pH 7.4 and 9.0, and their structural, mechanical, ion release, and antibacterial properties were determined. Results highlight pH‐dependent properties and demonstrate the potential of these hydrogels for bone tissue engineering applications.

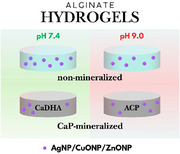

## 1 Introduction

Hydrogels are porous, three‐dimensional networks of hydrophilic polymers known for their high water absorption and low solubility in water [1, 2, 3]. Depending on the intended application, synthetic and natural polymers can be used for their preparation [4]. Natural polymers are commonly the polymers of choice for biomedical applications because of their good biological properties [5]. Among natural polymers, alginate, a linear polysaccharide obtained from brown algae or bacteria, attracts special attention. Composed of repeating units of *β*‐D‐mannuronic acid (M unit) and *α*‐L‐guluronic acid (G unit) linked by *β*‐1,4‐glycosidic bonds [6], alginate exhibits advantages including biocompatibility, low immunogenicity, low cost, mild gel formation conditions, and degradability [7, 8, 9, 10]. However, like many hydrogels, its poor mechanical properties and lack of temperature stability often restrict the potential for practical application [4].

To improve hydrogels’ properties and broaden the range of their applications, hydrogel composites with nanomaterials, so‐called nanocomposite hydrogels (NCHs), have recently attracted attention [4]. Since then, various nanoparticles (NPs), including carbon‐based, polymeric, metallic, and non‐metallic inorganic, have been used to prepare NCHs, resulting in materials with improved mechanical properties, electrical conductivity, magnetic, and biological properties [4]. Such NCHs are mainly investigated as imaging agents, drug delivery systems, wound dressings, cartilage implants, sensors, environmental protection materials, electronics, semiconductors, and ferromagnetics [4, 11, 12]. To date, alginate‐based NCHs with embedded metal or metal oxide NPs have been primarily investigated for wound therapy [7, 8, 13]. Alginate hydrogels are also of interest for bone tissue engineering, and efforts have been made to improve their mechanical properties by combining alginate with other polymers and nanostructures [14, 15].

NCHs with antimicrobial properties are of special interest as they are emerging as a promising solution for improving existing treatments of local and systemic infections without promoting antibiotic resistance, a growing global concern [11]. According to the World Health Organization, drug‐resistant bacteria cause an estimated 700 000 deaths worldwide each year [11]. Even more worrying is the prediction that the number of deaths will rise to 10 million per year by 2050 if no new treatments are developed [11, 16, 17]. Metal and metal oxide NPs, such as silver (AgNPs), copper oxide (CuONPs), and zinc oxide (ZnONPs), possess broad‐spectrum antibacterial properties and are less likely to induce resistance mechanisms [18]. When embedded in hydrogels, these NPs can be released in a controlled manner, reducing potential toxicity and improving local efficacy [11, 19, 20].

In addition to embedding antimicrobial NPs, another strategy to improve hydrogel performance is the mineralization of alginate with calcium phosphates (CaPs), the primary inorganic component of bone. This approach is of particular interest in bone tissue engineering as a way of mimicking the composition of bone [21, 22, 23]. Systematic studies on the effects of different NPs on the properties of bulk CaP‐mineralized alginate hydrogels are few. To the best of our knowledge, only Pogrebnjak et al. [24, 25] have investigated alginate hydrogels containing hydroxyapatite (HAP) and ZnO particles. These NCHs were obtained by mixing an alginate solution with a suspension containing ZnO particles and HAP and subsequent crosslinking with a calcium chloride solution. The hydrogel beads obtained showed pronounced antibacterial activity against both Gram‐negative and Gram‐positive bacteria.

The majority of previous studies on alginate hydrogels with incorporated CaPs or NPs were conducted using alginate beads and pre‐prepared CaPs and NPs. To the best of our knowledge, only Bjørnøy et al. [21] and our group [23] investigated CaP mineralization in bulk alginate hydrogels, while Lieu et al. [26] prepared multilayered hydrogels consisting of alternate alginate hydrogel and CaCO_3_ or CaP mineralized layers. In addition, the literature lacks data on comparing the influence of different antimicrobial NPs on biomaterial properties, which is of great importance for choosing the right NPs for different materials.

Motivated by this, this study aimed to prepare and characterize, for the first time, bulk in situ CaP‐mineralized alginate hydrogels incorporating the most frequently used antimicrobial NPs, namely silver (AgNPs), copper oxide (CuONPs), and zinc oxide (ZnONPs). Hydrogel formation and CaP mineralization were simultaneously initiated by the diffusion of calcium ions into phosphate‐containing alginate solutions in the presence or absence of NPs. Based on our previous work [23], two pH conditions were applied: pH 7.4, which favored the formation of predominantly crystalline CaP within hydrogels, and pH 9.0, which favored the formation of amorphous calcium phosphate (ACP) that was stable throughout the study [23], The NPs' concentration was chosen to enable the preparation of mineralized NCHs under the same conditions while minimizing the effect on the mineralization and gelation processes to a low extent. This approach allowed us to determine the best‐performing material for further studies in which its biological activity will be optimized.

## 2 Results and Discussion

**FIGURE 1:**
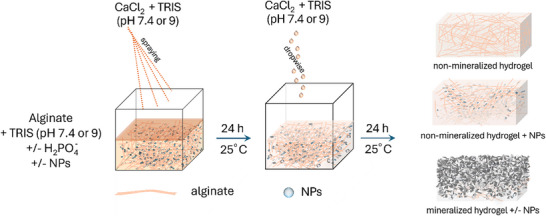
Scheme depicting the preparation of 1% alginate hydrogels. The hydrogels were prepared at pH 7.4 and 9.0 by dissolving the alginate and CaCl_2_ in TRIS buffer at the corresponding pH. The NPs were dispersed in alginate solutions. For mineralized hydrogels, the alginate solution also contained Na_2_HPO_4_. The formulations required to prepare hydrogels were dispensed from a syringe into silicone molds, and the surface was sprayed with CaCl_2_ solution using a fine‐spray bottle until the formulations were completely covered. After 24 h, the CaCl_2_ solution was removed, and a fresh one was added dropwise until the hydrogels were fully immersed. The gels were incubated for an additional 24 h at room temperature to complete crosslinking and mineralization.

Bulk, non‐mineralized and mineralized alginate hydrogels with or without incorporated Ag, CuO, and ZnO NPs were prepared by crosslinking with calcium chloride at pH values 7.4 and 9.0, as shown in Figure 1. To maintain the pH of the hydrogels, alginate and CaCl_2_ solutions were prepared in TRIS buffer at the specified pH. Depending on the type of hydrogel, Na_2_HPO_4_ was added to the alginate solution, and/or NPs were dispersed. The alginate solution was then dispensed into the molds and subsequently sprayed with CaCl_2_ solution using a fine‐spray bottle until the formulations were completely covered. The solution was applied by spraying to avoid disturbing the hydrogel surface. In this way, simultaneous crosslinking and mineralization were initiated in phosphate‐containing formulations. After 24 h, the CaCl_2_ solution was removed, and a fresh one was added dropwise until the hydrogels were fully immersed. The gels were incubated for an additional 24 h to complete the crosslinking and mineralization at 25 °C.

It is important to note that this is a diffusion‐limited process. Therefore, it is expected to create an inhomogeneous hydrogel with a mineralization gradient, likely to result in gradient mineralization, with a more heavily mineralized top surface.

The macroscopic appearance of the as‐prepared hydrogels was determined visually. Possible interaction of NPs and alginate was investigated by FTIR spectroscopy of freeze‐dried hydrogels, while the morphology of mineralized hydrogels was analyzed by SEM. For characterization of formed CaPs, the alginate was dissolved in citrate solution, and the extracted mineral precipitates were characterized by FTIR and PXRD. Rheological, ion‐release, and antibacterial studies were performed on as‐prepared hydrogels. For characterization of formed CaPs, the alginate was dissolved in citrate solution, and the extracted precipitates were characterized by FTIR and PXRD. Rheological, ion‐release, and antibacterial studies were performed on as‐prepared hydrogels. The characterization of NPs is shown in SI 1.

### 2.1 Characterization of Hydrogels

**FIGURE 2:**
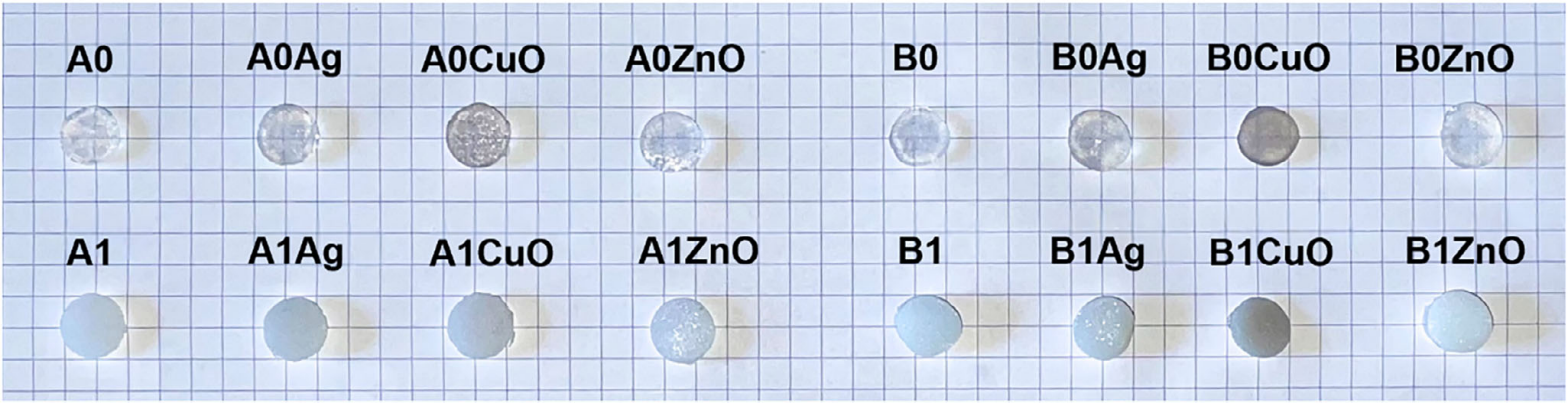
Macroscopic appearance of 1% alginate hydrogels, prepared at pH 7.4 (A) and pH 9.0 (B), non‐mineralized (A0, B0) and mineralized with calcium phosphate (A1, B1) incorporating Ag, CuO, or ZnO nanoparticles.

The macroscopic appearance of the cut synthesized hydrogels is shown in Figure 2. Non‐mineralized alginate hydrogels were transparent at both pH values. In contrast, hydrogels containing AgNPs were colored light gray, CuONPs brown, and ZnONPs white, which is in agreement with observations in previous studies [27]. All mineralized hydrogels were opaque, with a layer of CaP exposed on the surface of the hydrogels. In the case of the B1CuO hydrogel, the brown color was still visible. The visual observation of the cross‐section of mineralized hydrogels (Figure S1.1) revealed that mineralization was not limited to the surface. A mineralization gradient was observed, with only a thin bottom layer remaining unmineralized. Similar behavior was observed by Bjørnøy et al. [21] in the investigation of the formation and transformation of CaPs within the alginate hydrogel disks. It was observed that the disks' centers remained unmineralized, attributed to phosphate ion consumption and a reduction in pH. In recent years, gradient mineralized hydrogels have attracted attention as promising materials for treating osteochondral defects [28, 29], as they can mimic the complex hierarchical structure of the interface between articular cartilage and subchondral bone [30].

**FIGURE 3:**
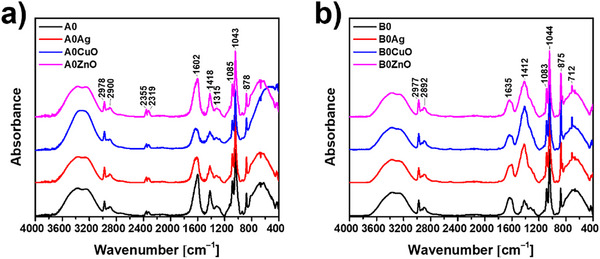
FTIR spectra of 1% alginate hydrogels, prepared at (a) pH 7.4 (A) and (b) pH 9.0 (B), non‐mineralized with calcium phosphates (A0, B0), incorporating Ag, CuO, or ZnO nanoparticles.

The Fourier Transform Infrared Spectroscopy (FTIR) spectra of the freeze‐dried, non‐mineralized hydrogels are shown in Figure 3. In the spectra of control hydrogels prepared at pH 7.4 and 9.0, broad bands in the 3700–3000 cm^−1^ region were observed, characteristic of water vibrations. The valence vibrations of the aliphatic C‐H groups were detected at 2978 and 2897 cm^−1^. In the spectra of the hydrogels prepared at pH 7.4, bands characteristic for the symmetric and asymmetric vibrations of the carboxylic anion (COO^−^) were observed at 1602 and 1418 cm^−1^, respectively. The band characteristic of *ν*(CCH) + *δ*(OCH) was observed at 1315 cm^−1^. The vibrations of the OCO group in the ring were detected at 1085 cm^−1^, while the stretching vibrations of C─O were detected at 1043 cm^−1^. The band characteristic of C1‐H deformation of mannuronic acid residues was observed at 878 cm^−1^ [31, 32, 33, 34]. No significant changes were observed in the FTIR spectra of hydrogels with CuONPs and ZnONPs, suggesting that there is little direct interaction between the NPs and the alginate monomers [31]. In the spectra of hydrogels prepared at pH 9.0, a difference was observed in the spectral features of the bands characteristic of the symmetric and asymmetric vibrations of the carboxylic anion (COO^−^). The position of the *ν*_(COO)sym_ band was shifted to 1620 cm^−1^, and the relative ratio of the intensities of these two bands was reversed compared to the spectra of hydrogels prepared at pH 7.4. In addition, the hyperfine structure of the band at 1412 cm^−1^ was lost in the hydrogels with NPs. This could indicate a stronger interaction between the NPs and the alginate monomers compared to the hydrogels prepared at pH 7.4.

The preparation of composite alginate hydrogels with a mineral phase has been proposed as a way to improve mechanical properties and stability [35]. Most of the work has been done with calcium phosphates and calcium carbonates to mimic the structure and composition of natural bone [21, 22, 23, 36, 37]. Recently, however, the incorporation of antimicrobial mineral phases, such as zinc [35] or copper phosphate [38], has also been investigated. Most of the work on the mineralization of alginate has so far been done on alginate beads [24, 35, 36, 37, 38]. Attempts have been made both to mix the pre‐prepared mineral phase [24, 38] and to form it in situ [22, 23, 35, 39].

**FIGURE 4:**
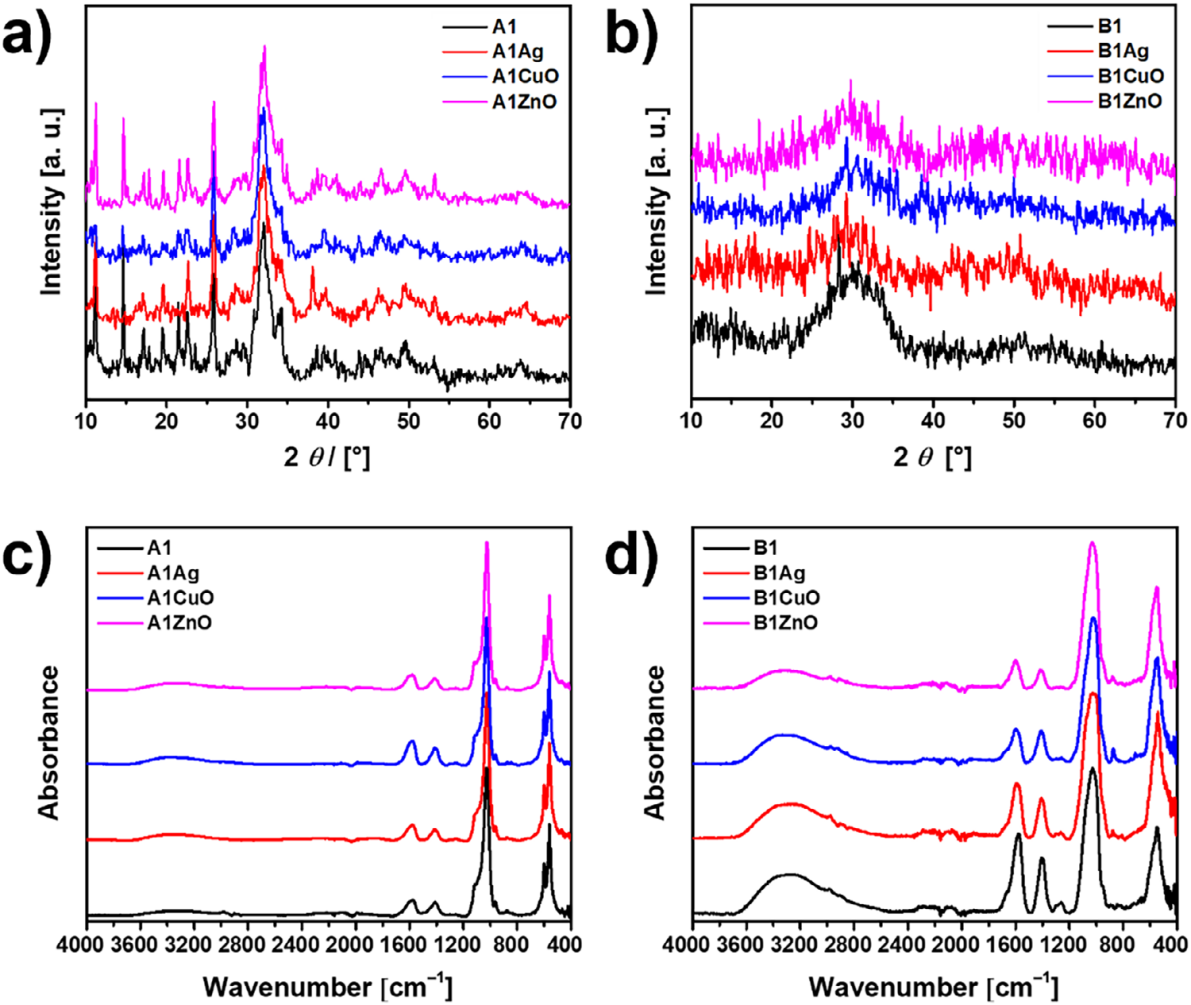
(a,b) PXRD patterns and (c,d) FTIR spectra of 1% alginate hydrogels prepared at pH 7.4 (a,c) and pH 9.0 (b,d), mineralized with calcium phosphates (A1, B1), incorporating Ag, CuO, or ZnO nanoparticles. A—hydrogels prepared at pH 7.4, B—hydrogels prepared at pH 9.0.

To obtain bulk mineralized gels, mineralization was initiated simultaneously with the gelling process by spraying a calcium chloride solution over an alginate solution in phosphate. Since mineralization is a faster process than gelation [36], the concentration of Na_2_HPO_4_ was chosen based on our previous work [23]. To determine the composition of the CaP formed, the mineralized hydrogels were treated with sodium citrate to remove the alginate and extract the precipitate for powder X‐ray diffraction (PXRD) and FTIR analysis (Figure 4). PXRD patterns of all mineralized samples contain characteristic apatitic peaks at 2*θ* 25.9° and 31.7°, as well as low intensity peaks at 2*θ* 46.6° and 49.5° characteristic of calcium‐deficient hydroxyapatite (CaDHA, JCPDS 00‐046‐0905). Peaks characteristic of AgNP (JCPDS 4‐0783) at 2*θ* 38.1°, 44.7°, and 64.6° were additionally observed in the pattern of the A1AgNP sample. Peaks characteristic of CuONPs and ZnONPs could not be unambiguously discerned.

The PXRD patterns of precipitates formed at pH 9.0 contained only a broad amorphous peak from 2*θ* 23.1° to 36.4°, revealing the formation of ACP [40, 41]. This is consistent with our previous studies [23] showing that the alginate hydrogels can be mineralized with different CaP depending on the pH. At pH 9.0, ACP formed and remained stable for a prolonged time.

In the FTIR spectra of mineralized hydrogels, bands characteristic of alginate and CaP were observed. The broad band at 3700–2700 cm^−1^ observed in all spectra is characteristic of the stretching vibration of the OH group, which can originate from both CaP and alginate. The bands at 1585 and 1419 cm^−1^ can be ascribed to symmetric and asymmetric stretching vibration of the carboxyl group in alginate [27]. In the spectra of the precipitates formed at pH 7.4, bands characteristic of the asymmetric stretching mode of ${\mathrm{PO}}_{4}^{3-}$ were observed at 1116 and 1026 cm^−1^. The band characteristic of ${\mathrm{PO}}_{4}^{3-}$ symmetric stretching was found at 996 cm^−1^, while bands observed at 598 and 561 cm^−1^ are characteristic for bending modes of ${\mathrm{PO}}_{4}^{3-}\;$ [42]. Spectra of precipitates formed at pH 9.0 contained bands characteristic of ACP, namely the asymmetric stretching mode of ${\mathrm{PO}}_{4}^{3-}$ at 1027 cm^−1^, ${\mathrm{HPO}}_{4}^{2-}$ band at 875 cm^−1^, bending mode of the ${\mathrm{PO}}_{4}^{3-}$ at 558 cm^−1^ [40, 41]. The lack of splitting of the bands at 1027 and 558 cm^−1^ is indicative of the formation of ACP [41]. From the PXRD and FTIR characterization, it can be concluded that NPs do not influence the composition of the forming mineral phase.

Comparing the obtained results with our previous work [23], a difference in the composition of CaP formed at pH 7.4 is observed. Previously, alginate hydrogels mineralized with octacalcium phosphate (OCP) were obtained by diffusion of Ca^2+^ into alginate solutions contained within dialysis membranes [23]. In this work, CaDHA was obtained by spraying the alginate solution with Ca^2+^ solution. A number of studies have shown that, due to structural similarity, OCP readily transforms to the apatitic phase [43]. Which of them forms under certain experimental conditions depends on the delicate interplay of thermodynamic and kinetic factors. The different diffusion rates of Ca^2+^ in these two studies are likely the cause of the observed differences in the composition of the formed phase. Previous studies on alginate bead mineralization have identified the Ca/P ratio and bead size as important factors in determining the CaP composition [22, 36, 44, 45].

However, no difference in the composition of CaP formed at pH 9.0 was observed in these studies, as ACP was formed and stable for a long time in both cases. The ACP is well known for its tendency to transform into more stable crystalline phases in aqueous environments if no stabilization factors are present [40, 41, 46, 47, 48]. The interplay of pH, presence of alginate, and confined environment can be a reason for stabilization of ACP in hydrogels prepared at pH 9. The influence of pH on ACP stability is complex. Previous studies have shown that the lifetime of freshly prepared ACP increases with increasing pH from 7.4 to 10.5, and then decreases, and at pH ∼12.8 is nearly the same value as at pH ∼7.4 [47, 49], confirming our results. Ucar et al. [50] have shown that alginate‐based additives can retard both ACP formation and transformation in solution. In addition, several studies have demonstrated that the ACP lifetime can be prolonged in confined environments [51, 52, 53].

**FIGURE 5:**
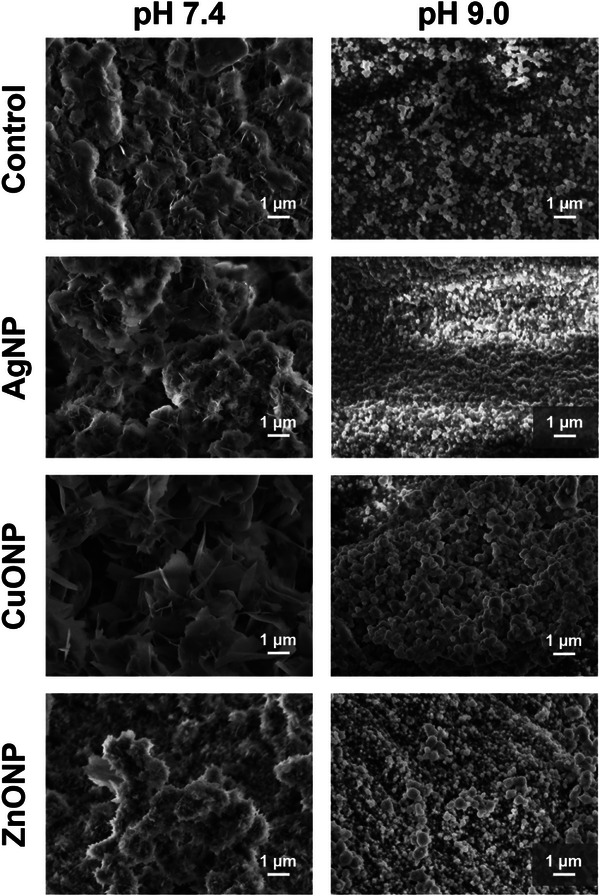
SEM of 1% alginate hydrogels, prepared at pH 7.4 and pH 9.0, mineralized with calcium phosphates, incorporating silver (AgNPs), copper oxide (CuONPs), or zinc oxide nanoparticles (ZnONPs).

Micrographs obtained with scanning electron microscopy (SEM) of hydrogels mineralized at pH 7.4 revealed the morphology of the crystals formed at the top surface of the hydrogels, i.e., at the site of the first contact with CaCl_2_ solution. The thin, irregular plate‐like crystals, characteristic of CaDHA [46] (Figure 5), were observed. The type of NPs incorporated into the hydrogel significantly influenced the morphology of the precipitates obtained. The largest and most developed crystals were obtained in the presence of CuONPs, while smaller and more aggregated crystals were formed in the presence of AgNPs and ZnONPs. Chain‐like aggregates of spherical particles characteristic of ACP [54, 55] were observed in the SEM micrographs of all hydrogels prepared at pH 9.0. As with the crystalline phase, the influence of the type of NPs on the size of the ACP particles was also observed. The smallest, 181.9 ± 32.5 nm, and least aggregated ACP particles were obtained in the presence of AgNP. Larger, polydispersed ACP particles were formed in the presence of CuONPs (371 ± 105 nm) and ZnONPs (331 ± 130 nm).

NPs used in this study differ in composition, active surface area, surface structure, and charge density, parameters of utmost importance for precipitation processes. Previous studies on the influence of differently stabilized AgNPs [56], as well as TiO_2_ nanomaterials of different morphologies [57] on CaP formation and transformation showed that these nanomaterials can influence the morphology of both amorphous and crystalline phases. In addition, AgNPs influenced the composition of the precipitate, as the amount of OCP formed in the mixture with DCPD decreased with increasing AgNPs concentration.

Introduction of a mineral phase into alginate hydrogels generally increases compressive strength, modulus, and elasticity [14, 58, 59, 60, 61]. The extent of improvement of mechanical properties depends on the amount of added inorganic phase. However, at least for alginate beads, it was independent of the type of CaP used [60]. The observed effects were ascribed to the partial dissipation of deformation energy by the formed layer of mineral phase [62, 63]. In this paper, the rheological properties of the alginate hydrogels were investigated to evaluate their mechanical response. The storage (*G*', elastic response) and loss (*G*'', viscous response) modulus were recorded as a function of frequency in frequency sweep tests [64, 65]. The storage modulus, *G*', displays a slight dependence on frequency in all formulations, highlighting the viscoelastic nature of the hydrogel, which is expected from ionically crosslinked alginate [66, 67, 68]. Up to the highest frequencies investigated, the elastic contribution is higher than the dissipative component (*G*' > *G*''), indicating gel‐like behavior for all investigated hydrogels [67].

**FIGURE 6:**
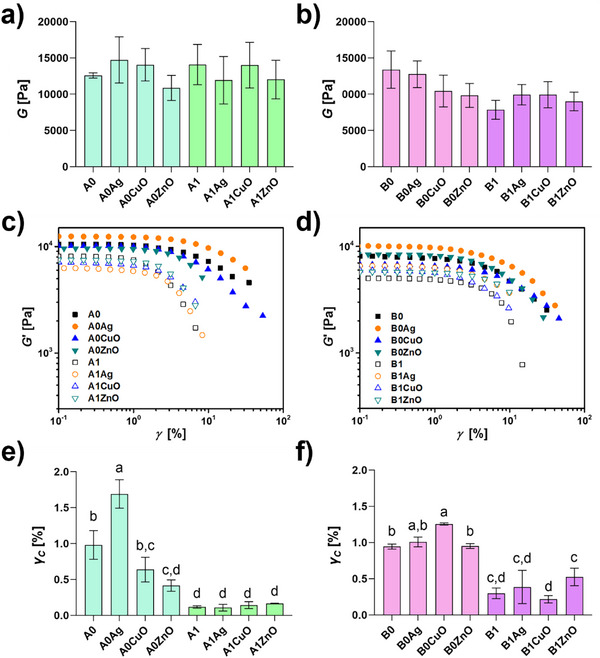
(a,b) Shear modulus, (c,d) stress sweep analysis showing storage modulus (*G'*) as a function of deformation (*γ*), and (e,f) shear stress of 1% alginate hydrogels, prepared at pH 7.4 (A) and pH 9.0 (B), non‐mineralized (A0, B0) and mineralized with calcium phosphates (A1, B1), incorporating Ag, CuO and ZnO nanoparticles. Error bars represent standard error. Means with different letters are significantly different. Full symbols—non‐mineralized samples, empty symbols—mineralized samples, *ϑ* =25 °C, *p* ≤0.05.

More specifically, the values of shear moduli (*G*) of the non‐mineralized hydrogels, as well as the mineralized hydrogels, determined via Equation 3 were 13.1 ± 4.8 kPa under all analyzed conditions (Figure 6a). Since the differences between them were not significant, the results indicate that the NPs at pH 7.4 did not influence the stiffness of the hydrogels. Previously, it was shown that incorporating AgNPs into alginate hydrogels results in slightly reduced *G*' values. However, as in our work, *G*' was higher than *G*'' across the tested frequencies (Figure S3.1) [69, 70].

Addition of ZnONPs to sodium alginate‐chitosan oligosaccharide hydrogel significantly enhanced the mechanical properties of the hydrogel, due to multiple interactions between the NPs and polymers [10]. It is worth noting that in these studies, at least a 20‐fold higher NPs concentration was used. Contrary to this, dispersing HAP and *β*‐tricalcium phosphate (*β*‐TCP) NPs in alkyl functionalized gellan gum derivative hydrogels resulted in an increase of *G*' with the concentration of the NPs [71]. Incorporated NPs can contribute to network reinforcement but may also inhibit the formation of crosslinking sites between alginate chains, potentially reducing the tension required to disrupt the elastic matrix, as was previously observed for AgNPs [69]. In our case, this effect was more pronounced in mineralized hydrogels.

Hydrogels prepared at pH 9.0 showed somewhat different behavior. No significant differences were observed in the *G* values of different hydrogels. However, the hydrogels prepared at pH 9.0 had an overall lower stiffness (9.5 ± 3.0 kPa) compared to the hydrogels prepared at pH 7.4, except B0 and B0Ag. It is reported that dissociation constants (p*K*_a_) for M and G unit monomers are 3.38 and 3.65, respectively [72], and that in aqueous solution with a pH range of 5–9, alginate behaves as a polyanion because the carboxylic groups in sodium alginate are fully dissociated [73]. In addition, in an alkaline medium, these dissociated, negatively charged carboxylate ions exert repulsive forces on one another, increasing water absorption and swelling of the hydrogel, which also negatively impacts gel strength [33, 74].

In addition to pH, it is also possible that the rheological properties of hydrogels were affected by the release of Cu^2+^ and Zn^2+^ ions, which could replace Ca^2+^ ions in the eggbox structure due to their higher affinity for the polymer. Previous studies have shown that the type of crosslinker ion can influence the mechanical properties of alginate hydrogels. Agulhon et al. [75] investigated the influence of Co^2+^, Mn^2+,^ and Cu^2+^ ions on the mechanical properties of alginate gels with different guluronic acid content. All hydrogels behaved as viscoelastic solids, but in the case of the hydrogels crosslinked with Cu^2+^, the flow zone was not observed. In addition, the Cu^2+^ crosslinked hydrogels were stiffer compared to the other gels, with *G*' always showing values above 10^4^ Pa. Similarly, Malektaj et al. [76] observed that the values of *G*' and *G*'' of alginate hydrogels crosslinked with different multivalent cations, decrease in the order Cu‐alginate > Sr‐alginate > Ca‐alginate > Zn‐alginate.

According to Papageorgiou et al. [77] and Falcone et al. [78], metal‐alginate complexes have a pseudo‐bridged unidentate arrangement. In such a case, if no significant difference is observed in the wavenumbers of the symmetric and asymmetric FTIR vibrations of the carboxylic anion in different materials, as in our case, it can be considered that the complexes with a similar structure are formed [78].

The non‐linear response of the gels to stress was evaluated with stress sweep experiments. The results of the analysis, which show the dependence of the storage modulus (*G*') on the deformation (*γ*) (Figure 6c,d), highlight the presence of a strain softening behavior in all formulations. However, statistically significant differences in the critical strain values, i.e., the value of deformation at which strain softening sets in, were observed among different hydrogels (Figure 6e,f). Compared to A0, the hydrogels prepared at pH 7.4 show an early breakdown of the network, except for the A0Ag hydrogel. This difference is particularly evident for the mineralized hydrogels (A1, A1Ag, A1ZnO, A1CuO). Contrary to this, the non‐mineralized hydrogels prepared at pH 9.0 showed similar or delayed network breakdown, with B0Ag and B0Cu being the most resistant. In contrast, their mineralized counterparts exhibit an earlier exit from the non‐linear regime, resulting in a lower critical strain. Indeed, the critical strain values were greater for mineralized hydrogels prepared at pH 9.0 than for those prepared at pH 7.4. The difference could be a consequence of the difference in properties of the mineral phases formed at different pH. However, it is essential to note that a direct correlation between the surface microstructure observed in SEM (Figure 4) and the bulk mechanical properties (Figure 5) is complex due to the inherent inhomogeneity of the mineralized samples. The sample preparation resulted in a gradient mineralized hydrogel with a heavily mineralized, stiff top surface and a thin bottom non‐mineralized layer. Consequently, the SEM analysis characterizes the local, micro‐scale morphology of the top surface, while the rheological measurements capture the bulk, average mechanical response of the entire, inhomogeneous sample. The bulk properties are therefore likely dominated by this macro‐scale structural gradient rather than by the specific micro‐scale crystal morphologies.

**FIGURE 7:**
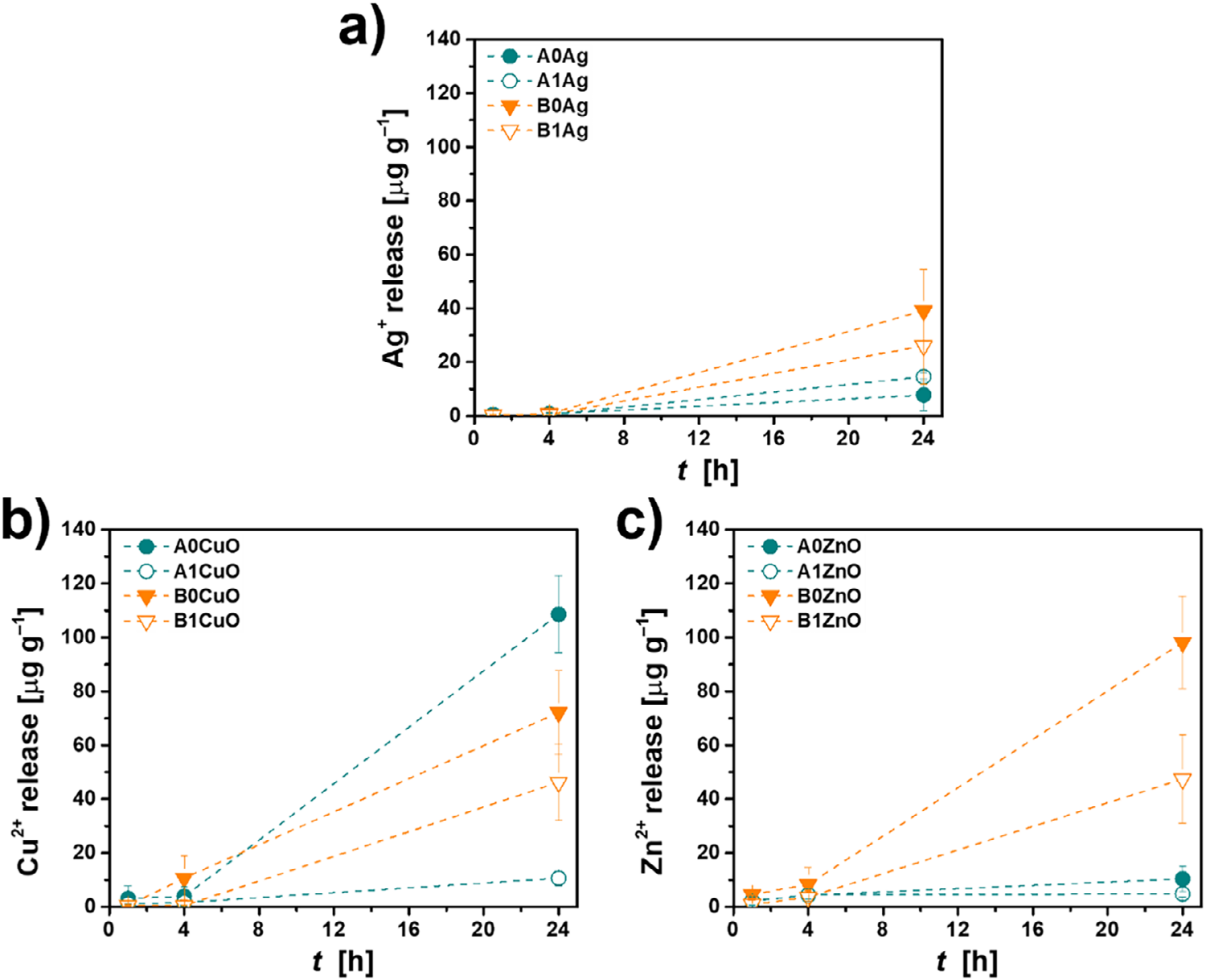
Release of (a) Ag^+^, (b) Cu^2+^ and (c) Zn^2+^ during 1, 4, and 24 h from 1% alginate hydrogels, prepared at pH 7.4 (A) and pH 9.0 (B), non‐mineralized (A0, B0) and mineralized with calcium phosphates (A1, B1), incorporating Ag, CuO and ZnO nanoparticles in phosphate buffer saline at 37 °C, pH = 7.4. The lines are only guidelines for the eye.

Different trends in the release of different ions were observed depending on the pH at which the hydrogel was prepared and mineralized (Figure 7). In the case of hydrogels with AgNP, more silver was released from the ones prepared at pH 9.0 (Figure 7a). In the case of hydrogels containing CuONPs, more copper was released from non‐mineralized hydrogels (Figure 7b), while in the case of ZnONPs, more zinc was released from non‐mineralized hydrogels compared to mineralized ones and from hydrogels prepared at pH 9.0 compared to those prepared at pH 7.4 (Figure 7c). In all cases, release was slower in the first 4 h than in the later stages, in contrast to previous observations for the release of Zn [35] and Sr [79]. However, a similar behavior was observed for the release of Cu^2+^ from calcium crosslinked alginate microbeads containing prepared copper minerals with phosphate reagent in Dulbecco's Modified Eagle Medium (DMEM) [38].

The hydration and disintegration capacity in PBS at 37 °C were also determined (Table 1). At pH 7.4, only mineralized hydrogels with incorporated NPs were stable, and non‐mineralized one with incorporated AgNPs. The degree of hydration varied depending on the type of NP, increasing in the order: A1Ag, A1CuO, and A1ZnO. At pH 9.0, the non‐mineralized hydrogels showed a different trend, with B0Ag exhibiting a significantly higher hydration degree compared to B0CuO. Mineralized hydrogels showed different behavior at pH 9.0, with B1CuO showing slightly higher hydration than non‐mineralized B0CuO and B1ZnO showing the highest degree of hydration. Hydrogels for which no values were recorded (A0, A0Ag, A0CuO, A0ZnO, A1, B0, B0ZnO, B1, and B1Ag) had their structures completely broken down, leading to disintegration in the PBS. This is in accordance with a study by Qian et al. [80], in which it was shown that the degradation of alginate hydrogels is accelerated in the presence of PBS and at higher temperatures.

**TABLE 1:** The hydration and disintegration capacity of 1% alginate hydrogels when exposed to phosphate buffer saline for 24 h at 37°C. Hydrogels were prepared at pH 7.4 (A) and pH 9.0 (B) as non‐mineralized (A0, B0) and mineralized with calcium phosphates (A1, B1), incorporating Ag, CuO, and ZnO nanoparticles.

Sample^a^	Hydration degree
A1Ag	15.1 ± 4.8%
A1CuO	47.0 ± 19.4%
A1ZnO	53.9 ± 1.8%
B0Ag	88.7 ± 9.4%
B0CuO	41.1 ± 2.2%
B1CuO	53.5 ± 2.4%
B1ZnO	67.0 ± 13.0%

^a^ For hydrogels A0, A0Ag, A0CuO, A0ZnO, A1, B0, B0ZnO, B1, and B1Ag, the values were not recorded as samples were completely disintegrated, losing their hydrogel structure.

Literature data on the influence of NPs addition on alginate hydrogels' swelling behavior are scarce and not comparable due to the different media applied. Previous studies have shown that adding ZnONPs can reduce the degree of swelling, which was attributed to the decrease of available volume within the hydrogel network [81]. On the other hand, it was also observed that the addition of ZnONPs did not affect the swelling of the sodium alginate/chitosan composite hydrogel in a wound simulation solution [10]. However, the addition of ZnO to hydroxyapatite‐alginate composites increased the swelling in PBS [24].

### 2.2 Antibacterial Activity

A variety of nanomaterials, including metal/metal oxide NPs, are being intensively investigated as a promising solution to antimicrobial resistance. Nanomaterials can simultaneously induce various damages to bacterial cells, including disruption of cell membranes, inhibition of quorum sensing, ATP depletion, membrane dysfunction, disruption of intracellular processes, inactivation of enzymes essential for metabolic processes, and damage to lipids, proteins, and nucleic acids [82]. Metal/metal oxide NPs can exhibit antibacterial activity not only in direct contact with bacterial cells but also through released ions that can affect bacteria surrounding the implant, thereby preventing infection [83].

**FIGURE 8:**
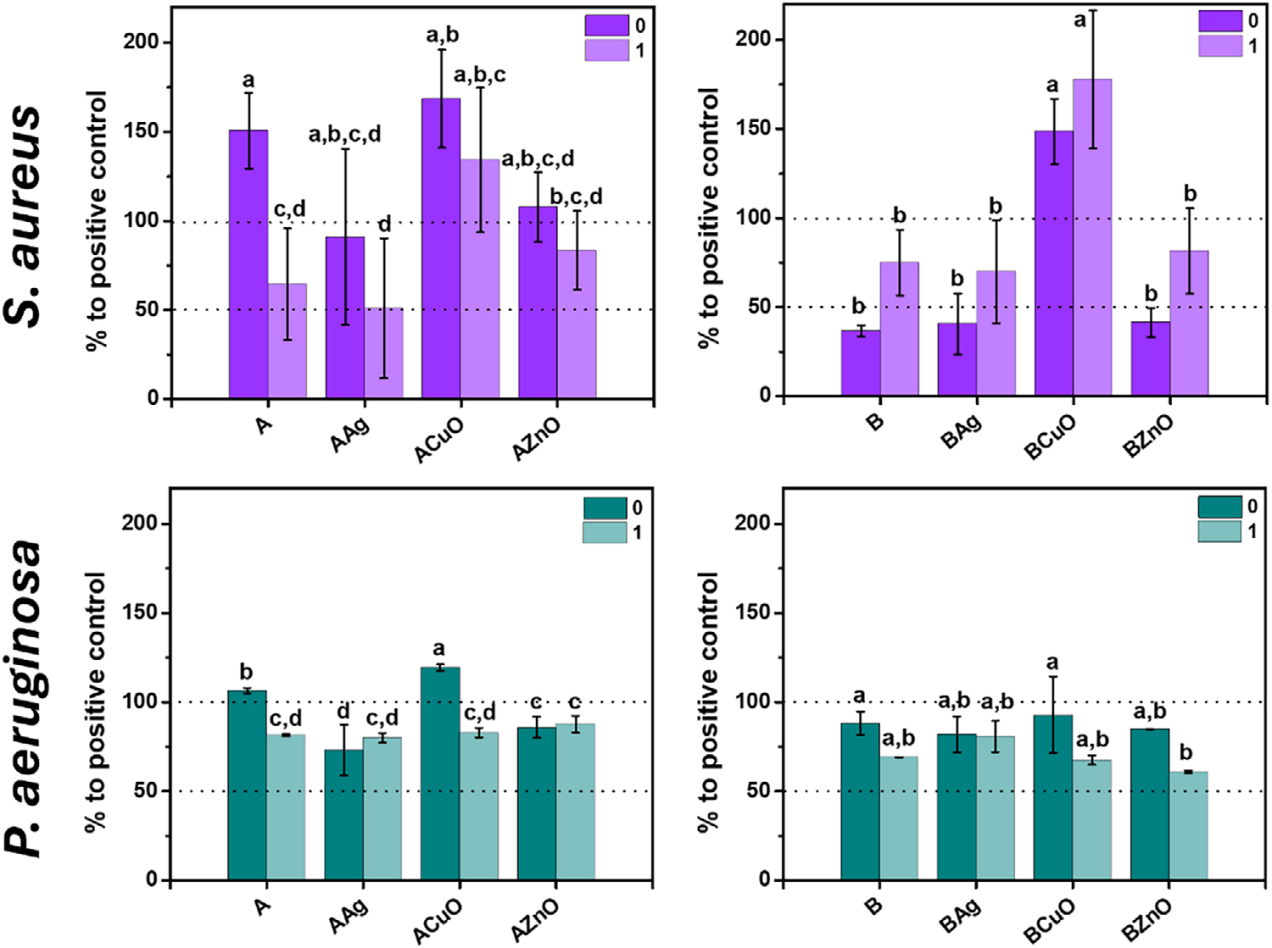
Planktonic growth expressed as percentage of growth relative to positive control of *Staphylococcus aureus* and *Pseudomonas aeruginosa* with 1% alginate hydrogels, prepared at pH 7.4 (A) and pH 9.0 (B), non‐mineralized (A0, B0) and mineralized with calcium phosphates (A1, B1), incorporating Ag, CuO, and ZnO nanoparticles. Error bars represent standard deviation. Means with different letters are significantly different, *p* ≤0.05.

Screening of the hydrogels for antibacterial activity was performed using the OD600 planktonic growth assay of two bacterial strains commonly associated with implant infections, namely *S. aureus* and *P. aeruginosa*, were investigated. The bacterial growth is expressed as a percentage of the growth of the positive control (Figure 8).

The planktonic growth of *S. aureus* was markedly dependent on the pH at which hydrogels were prepared, as well as on the mineralization of the hydrogels. In general, except for hydrogels containing CuONPs, the planktonic growth of *S. aureus* was lower in the presence of hydrogels prepared at pH 9.0. The dominant mechanism of growth inhibition is likely that the hydrogels raised the medium pH, thereby inhibiting planktonic growth, in accordance with previous observations that planktonic growth was slightly reduced during the early growth phase (3–6 h) at pH 8.5 [84]. In this study, however, the reduced growth was still observed after 24 h.

The difference in effect on planktonic growth between non‐mineralized and mineralized hydrogels prepared at pH 7.4 was observed. In all cases, planktonic growth was higher for non‐mineralized hydrogels than mineralized ones, the difference being statistically significant only for the alginate hydrogel. Similar inhibition of bacterial growth on CaP‐mineralized surfaces was observed in our previous work on magnetron‐sputtered surfaces [85].

It is worth noting that the growth of *S. aureus* was enhanced in the presence of all hydrogels containing CuONPs. The percentage of planktonic growth ranged from 134.2 ± 40.5% for A1CuO to 177.8 ± 38.8% for B1CuO. Previous study has shown that the growth of *S. aureus* was stimulated by copper doses of up to 1 mm, especially in TSB media [86], the same media used in this study. There is also evidence that certain strains can survive and grow in copper‐rich environments through their resistance mechanisms [87]. Stimulated growth of *S. aureus* was also observed with A0 hydrogel (150.6 ± 21.3%).

The effect of pH and mineralization on the planktonic growth of *P. aeruginosa* was much more subtle. The planktonic growth was statistically significantly higher in the presence of A0 and A0CuO hydrogels compared to all other hydrogels. At pH 9.0, a statistical difference was observed between B and B0CuO, on one side, and B1ZnO on the other. Slightly increased planktonic growth of *P. aeruginosa* was observed in the presence of A0CuO (106.5 ± 1.0%). However, mineralization with CaP reduced the growth of *S. aureus* in the presence of all hydrogels prepared at pH 7.4. Significant reduction of the growth was observed in the presence of A1 (64.5 ± 31.2%).

Rescignano et al. [70] tested AgNP‐containing hydrogels and were able to demonstrate inhibition of both *Escherichia coli* and *P. aeruginosa*. However, they used an AgNP concentration of 2.5%, 5%, and 7.5% (w/w), which is 500 times higher than our initial concentration. Another study showed that an AgNP‐containing alginate‐chitosan hydrogel also inhibited *E. coli* and *Bacillus subtilis* using the zone‐inhibition test [88]. In this case, the AgNPs were synthesized in situ, and the concentration of AgNPs in the hydrogel was not reported. AgNPs were also incorporated into hydrogels made of polyvinyl alcohol and alginate. In that case, a hydrogel containing polyvinyl alcohol and alginate at a 9:1 ratio and a 120 mm Ag+ concentration showed the best performance, with survival rates of 1.6% for S. aureus and 4.5% for *E. coli* [89].

Johari et al. [90] incorporated CuNPs into chitosan‐alginate hydrogels at concentrations of 2%, 3.5%, and 5% (w/w), with a concentration‐dependent effect, whereby 5% showed the strongest antibacterial activity against both *E. coli* and *S. aureus* using the zone inhibition test. Cleetus et al. [91] investigated 3D‐printed alginate hydrogels infused with ZnONPs for antibacterial applications and showed that 1% (w/v) ZnONPs exhibited significant antibacterial activity against *Staphylococcus epidermidis*.

Development of biomaterials with antibacterial properties requires a balance between antibacterial efficacy and biocompatibility. Previous studies have demonstrated that the antibacterial mechanisms of NP‐incorporated hydrogels typically involve contact killing, release of metal ions, and generation of reactive oxygen species (ROS). The contact‐based mechanism was emphasized in the study by Cleetus et al. [91], in which they suggested that the small size of particles (4‐6 nm) allowed close contact that disrupted the bacterial cell membrane and caused cell leakage. In addition, ROS formation was observed, especially when ZnONPs were exposed to UV light, thereby enhancing the antibacterial effect through the production of hydroxyl radicals. In the study by Johari et al. [90], the ion release of Cu^2+^ was emphasized as the primary mechanism. Razi et al. [92] compared ZnONPs and Zn hydroxyacetate NPs in alginate hydrogels and demonstrated that Zn‐hydroxyacetate was more effective due to its higher solubility, resulting in a greater release of ions. Yadollahi et al. [93] investigated CuONPs in carboxymethylcellulose hydrogels and attributed the mechanism of action to a combination of ion release and ROS generation.

In this study, the effect on planktonic growth appeared linked to the specific ion release profiles. For example, the Ag‐incorporated hydrogels exhibited the lowest ion release, consistent with their lower impact on planktonic growth compared to pure hydrogels. Conversely, despite variations Cu^2+^ release, *S. aureus* growth was pronounced in all CuONP‐containing hydrogels. This occurred even in hydrogels prepared at pH 9.0, which otherwise inhibited *S. aureus* in all other formulations. This observation requires further investigation to elucidate the underlying mechanism. Meanwhile, the A0ZnO hydrogel, which exhibited the highest Zn^2+^ release, resulted in slight inhibition of *P. aeruginosa*.

Alginate itself is considered a biocompatible biomaterial [69, 94]. As mentioned, the concentration of NPs in this study was lower than those frequently reported in the literature. While increased concentration is expected to enhance antibacterial performance, it can also increase cytotoxicity, an important parameter for future applications. Thus, it was previously demonstrated that incorporation of AgNPs into an alginate hydrogel results in concentration‐dependent toxicity toward Vero cells [69], whereas incorporation of ZnONPs results in low toxicity toward NIH‐3T3 fibroblasts and human kidney epithelial cells [10]. Crosslinking alginate hydrogel with copper ions resulted in the stimulation of NIH‐3T3 fibroblasts without eliciting an inflammatory response [15]. In contrast, it was shown that the cytotoxicity of chitosan hydrogels crosslinked with Cu^2+^ and Zn^2+^ depends on the copper content [95]. In addition, the literature data indicate that cytotoxicity results are method‐dependent, as, for example, concentrations that were highly toxic to chondrocytes in conventional 2D monolayer cultures were nontoxic when cells were cultured in 3D perfusion bioreactors under biomimetic conditions [7].

In further optimization, the effect of increased NPs concentration on gelation and mineralization should not be neglected, as it can have a more profound impact on physico‐chemical properties [56, 57], thereby affecting the potential for targeted application and biological activity.

## 3 Conclusions

In the search for an ideal bone substitute material, composite materials consisting of inorganic materials dispersed within a hydrogel matrix are attracting attention, as their composition mimics the structure of bone. In particular, CaP‐containing hydrogels have been widely studied for their potential in bone tissue engineering, with various strategies explored to improve their biomimetic properties. In this sense, incorporating antibacterial agents into hydrogel matrices represents a promising strategy for local infection control.

This study demonstrated that the incorporation of metal and metal oxide NPs (AgNPs, CuONPs, ZnONPs) into alginate hydrogels, both non‐mineralized and CaP‐mineralized, can affect their structural, mechanical, and antibacterial properties. Using a simultaneous crosslinking and mineralization approach, mineralized NCHs were successfully prepared at two pH values, yielding poorly crystalline CaP (CaDHA) at pH 7.4 and stable amorphous CaP (ACP) at pH 9.0. Although NPs did not alter the overall composition of CaP phases, they have affected the morphology and size of the formed crystals, especially in the case of CaDHA.

The NPs had no significant effect on the rheological properties of the hydrogels. A particular difference was observed in the critical strain values between the mineralized and non‐mineralized hydrogels, with the latter showing an especially evident early network breakdown. The release of ions has shown dependence on mineralization and pH. Generally, higher ion release was observed for non‐mineralized hydrogels and those prepared at pH 9.0. In the initial 4 h, the release exhibited slower kinetics, which was followed by an accelerated release.

The antibacterial efficacy of the hydrogels varied with pH, mineralization, and NPs type. At pH 7.4, mineralized hydrogels, particularly A1 and A1Ag, significantly inhibited *S. aureus* growth. Conversely, at pH 9.0, non‐mineralized hydrogels like B0, B0Ag, and B0ZnO were more effective, possibly due to *S. aureus*'s sensitivity to alkaline conditions. For *P. aeruginosa*, differences were less pronounced, but mineralized hydrogels at pH 7.4, such as A1, A1Ag, and A1CuO, showed higher inhibition rates, while at pH 9.0, B1ZnO was the most effective. Importantly, the antibacterial effects observed in this study were achieved at lower NPs concentrations than those reported in previous studies. Interestingly, increased planktonic *S. aureus* growth was observed in the presence of all hydrogels containing CuONPs.

Overall, the results highlight how tuning formulation parameters, such as pH, mineralization, and NP type, is essential for optimizing structural integrity and antimicrobial performance. This study provides insights into the design of multifunctional hydrogels with potential applications in bone tissue engineering and infection control. Used as materials for treating smaller, non‐load‐bearing, irregular defects, they should support bone regeneration and reduce the probability of infections. Future work should focus on evaluating cytocompatibility and in vivo performance to further assess their potential for treating bone and osteochondral defects.

## 4 Experimental Section

### 4.1 Materials

Analytical grade chemicals calcium chloride dihydrate (CaCl_2_ · 2H_2_O, Sigma–Aldrich, Germany), sodium hydrogenphosphate (Na_2_HPO_4_, Sigma–Aldrich), Tris(hydroxymethyl)aminomethane (TRIS buffer, VWR Bdh Chemicals, UK), sodium citrate dihydrate (Na_3_C_6_H_5_O_7_ · 2H_2_O, Sigma–Aldrich, Germany), sodium alginate (Alginex grade L, Kimica Corporation, Japan), hydrochloric acid (HCl, Sigma–Aldrich), sodium hydroxide (NaOH, Sigma–Aldrich), nitric acid (HNO_3_, 65%, Suprapur, Merck, USA), multielement reference standard (Analytika, Czechia), phosphate buffer saline (PBS, Sigma–Aldrich), silver standard solution (Fluka, USA), silver nanopowder (20‐40 nm, Thermo Fisher Scientific USA), copper (II) oxide nanopowder (<50 nm, Sigma–Aldrich), and zinc oxide nanopowder (<50 nm, Sigma–Aldrich) were used in experiments.

For bacterial assays, *Staphylococcus aureus* DSM 1104 and *Pseudomonas aeruginosa* DSM 22644 were used, along with Tryptic soy agar (TSA, Biolife, Italy), Tryptic soy broth (TSB, Biolife), API Suspension Medium (bioMérieux, Marcy‐l'Étoile, France), D‐(+)‐glucose (Sigma Aldrich), and phosphate buffer saline (PBS, Sigma–Aldrich).

In all experiments, ultrapure water (UPW, conductivity 0.5 µS cm^−1^, Hydrolab HLP 10 UV, Hydrolab, Poland) was used.

### 4.2 Preparation of Stock Solutions and Suspensions

Buffered stock solutions (1 mol L^−1^ TRIS, 0.5 mol L^−1^ Na_2_HPO_4_, 0.2 mol L^−1^ CaCl_2_) were prepared by weighing chemicals, which were dried for 1 h at 80 °C. The pH of the solutions was adjusted to 7.4 or 9.0 with HCl or NaOH. A 2% alginate stock solution was prepared by dissolving the required amount of sodium alginate in UPW and heating at 70–80 °C with vigorous stirring for at least 1 h.

Nanoparticle stock suspensions (1000 mg L^−1^) were prepared by dispersing AgNP, CuONP, and ZnONP nanopowders in UPW, followed by sonication in an ultrasonic bath with ice for 20 min.

These stock solutions were combined to achieve the desired final compositions of hydrogels.

### 4.3 Preparation of Hydrogels

For non‐mineralized hydrogels, the final formulation contained 1% (w/v) alginate in 0.1 mol L^−1^ TRIS buffer at either pH 7.4 (A0) or pH 9.0 (B0), with or without 50 mg L^−1^ of AgNPs, CuONPs, or ZnONPs.

For mineralized hydrogels, the final formulation contained 1% (w/v) alginate in 0.1 mol L^−1^ TRIS buffer supplemented with 50 mmol L^−1^ Na_2_HPO_4_ at pH 7.4 (A1) or 25 mmol L^−1^ Na_2_HPO_4_ at pH 9.0 (B1), with or without 50 mg L^−1^ AgNPs, CuONPs, or ZnONPs.

The formulations were dispensed from syringes into the silicone molds (3 × 3 × 1.5 cm), and the surface was gently sprayed with 0.2 mol L^−1^ CaCl_2_, buffered to the same pH, using a fine‐spray bottle until the formulations were completely covered. This initiated simultaneous crosslinking and mineralization. After 24 h, the CaCl_2_ solution was removed, and fresh CaCl_2_ solution was added dropwise until the gels were fully immersed (approximately 3‐4 mL per sample). The gels were incubated for an additional 24 h to complete the crosslinking and mineralization at room temperature.

### 4.4 Powder X‐Ray Diffraction

To determine the average composition of the CaP formed within the samples, the mineralized hydrogels were treated with sodium citrate to extract the formed CaPs. The hydrogels were immersed overnight in 30 mL 0.2 mol L^−1^ sodium citrate at 25 °C, i.e., until the alginate dissolved. The resulting suspensions were centrifuged at 7500 rpm for 20 min. The supernatant was discarded, and the remaining precipitate was first washed with 30 mL UPW by centrifugation (15 min, 7500 rpm) and then with ethanol. The supernatant was discarded, and the remaining precipitate was dried under a stream of nitrogen and stored at 4°C until further analysis.

The PXRD patterns of the precipitates were obtained using the PANalytical Aeris Research Edition (Malvern PANalytical, UK) in Bragg‐Brentano geometry with CuK_α_ radiation. The angular scan range was from 3° to 70° 2*θ*, with a step size of 0.02 ° 2*θ* and a scan rate of 1°min^−1^.

### 4.5 Fourier Transform Infrared Spectroscopy

For FTIR analysis, the non‐mineralized hydrogels were freeze‐dried with FreeZone 2.5 (Labconco, USA) before analysis, while the CaPs precipitates from mineralized hydrogels were extracted by the same procedure used to prepare the samples for PXRD analysis.

FTIR spectra were recorded using a Tensor I FTIR spectrometer equipped with an attenuated total reflectance (ATR) module (Bruker, Ettlingen, Germany) in the range from 4000 to 400 cm^−1^ with a resolution of 1 cm^−1^. Each spectrum represents the average of 16 scans.

### 4.6 Scanning Electron Microscopy

The morphology of the freeze‐dried mineralized hydrogels and NPs was examined using a JEOL JSM‐7000F field emission scanning electron microscope, operating at 5 kV.

### 4.7 Transmission Electron Microscopy

For TEM analysis, a drop of the NPs suspension was placed onto a copper grid with the hollow Formvar membrane. Excess solution was removed with filter paper, and the grid was washed three times with a drop of UPW. After removing excess water, samples were dried under a nitrogen stream and stored in a desiccator in the dark until analysis.

Micrographs were acquired using a JEOL JEM‐1400Flash HC transmission electron microscope (JEOL, Tokyo, Japan) operated at 80 kV.

The sizes of NPs were determined using ImageJ 1.48v software (freely available at http://imagej.nih.gov/ij/) with at least 30 particles measured per sample.

### 4.8 Rheological Measurements

For the rheological measurements, the hydrogels were cut with a Ø = 20 mm punch (Matador, Germany) and analyzed with the HAAKE MARS III rheometer (Thermo Fisher Scientific, Dreieich, Germany). The tests were performed under oscillatory conditions to evaluate the mechanical properties of the hydrogels. First, the gap between the plates was adjusted with short stress sweep tests (*ν* = 1 Hz; stress range 1–5 Pa) until a consistent storage modulus (*G′*) was observed across all data points. After gap optimization, frequency sweep tests (*τ* = 1 Pa, *ν* = 0.01–100 Hz) and stress sweep (*ν* = 1 Hz, 1 Pa < *τ* < 10,000 Pa) were carried out at a temperature of 25 °C. To prevent water evaporation during the tests, a humidified environment was maintained by placing wet paper cloths in the sample hood (Thermo Fisher, Karlsruhe, Germany).

The shear moduli of the hydrogels were obtained by frequency sweep experiments with further analysis via the Maxwell model [66]. The storage and loss moduli were fitted as a function of angular frequency *ω* using the Equations 1 and 2, respectively, representing a series of spring dashpots connected in parallel and a purely elastic spring (*G*_e_):

(1) $$G^{\prime }=G_{e}+\sum_{i=1}^{n}G_{i}\frac{{\left(\lambda_{i}\omega \right)}^{2}}{1+{\left(\lambda_{i}\omega \right)}^{2}};\;G_{i}=\eta_{i}/\lambda_{i}$$

(2) $$G^{\prime \prime }=\sum_{i=1}^{n}G_{i}\frac{\lambda_{i}\omega }{1+{\left(\lambda_{i}\omega \right)}^{2}};\;G_{i}=\eta_{i}/\lambda_{i}$$

where *n* is the number of Maxwell elements used for the fitting. *G*_i_, *η*_i,_ and *λ*_i_ are the spring constant, the dashpot viscosity, and the relaxation time of the *i*‐th Maxwell element, respectively. The number of Maxwell elements taken into consideration was optimized by a statistical procedure reducing the *X* ^2^ · *N*_p_, where *X* ^2^ is the sum of the squared errors and *N*_p_(2+*n*) is the number of Maxwell elements. The relaxation times were considered dependent on each other and arbitrarily scaled by a factor of 10 [96]. The shear modulus was then calculated by Equation 3 and represents the shear modulus of gels under constant stress at small deformations:

(3) $$G=G_{e}+\sum_{i=1}^{n}G_{i}$$

The critical strain γ_*c*_ of the hydrogels were determined by fitting the experimental values with Equations 4 and 5 [97]:

(4) $$\sigma =\frac{G_{0}}{1+b\gamma }\gamma$$

where *G_0_* is the shear modulus for $\gamma_{\to 0}$ and *b* is a fitting parameter. Then, the critical strain was arbitrarily determined as:

(5) $$\gamma_{c}:\;\frac{\sigma }{G_{0}\cdot \gamma }=0.95$$

### 4.9 Inductively Coupled Plasma Mass Spectrometry (ICP‐MS)

To assess ion release, the hydrogels containing the NPs were immersed in 5 mL of PBS at 37 °C in static conditions in a water bath. Aliquots were taken after 1, 4, and 24 h, each aliquot being replaced with fresh PBS. Prior to analysis, the collected samples were diluted 100‐fold and acidified with 2% (v/v) HNO_3_. The total concentrations of Ag, Cu, and Zn were measured using the Agilent 8900 ICP‐QQQ instrument (USA). Elemental concentrations were determined by external calibration with standard solutions prepared by diluting a multi‐element reference standard (100 ± 0.2 mg dm^−3^) containing Cu and Zn, and a single‐element standard of Ag solution (1000 ± 0.002 g dm^−3^). The quality of measurements was ensured by simultaneous analysis of blank samples and internal control samples. Samples were analyzed in triplicate.

### 4.10 Hydration and Disintegration Capacity of Hydrogels

Alginate hydrogels containing NPs, used in release studies in PBS were also evaluated for stability to assess their hydration and disintegration capacity. The hydrogels were immersed in PBS at 37 °C for 24 h. Before immersion, the initial weight of each hydrogel (*W*_0 h_) and the weight after 24 h (*W*_24 h_) were recorded. The change in weight after 24 h was calculated using Equation 6 [98, 99]:

(6) $$\Delta Weight=\left(\frac{W_{24\;h}-W_{0\;h}}{W_{0\;h}}\right)\cdot 100$$

The results were normalized to the wet mass of each hydrogel and expressed as the cumulative mass of released metal per gram of hydrogel (µg g^−1^).

### 4.11 Antibacterial Characterization

Planktonic growth of *S. aureus* and *P. aeruginosa* on hydrogels was evaluated. Before testing, hydrogels were immersed in 70% ethanol for 20 min and then washed with sterile UPW for sterilization [100]. Negative controls have shown that hydrogels remain sterile for up to four days using this method.

Bacterial inocula were prepared from fresh cultures grown overnight on tryptic soy agar. These cultures were adjusted to 0.5 MacFarland in 5 mL of 0.85% API Suspension Medium and then diluted in TSB supplemented with 1% glucose to a final concentration of 5 · 10^4^ CFU mL^−1^. The inoculated samples were placed in 24‐well microplates and incubated overnight at 35 °C aerobically. After 24 h, 100 µL aliquots were transferred to 96‐well plates and the absorbance at 600 nm (OD_600_) was measured using an Infinite 200 PRO microplate reader (TECAN, Grödig, Austria). All experimental runs used negative (media only) and positive (media and bacteria only; no sample) controls and were performed with biological and technical replicates.

### 4.12 Statistical Analysis

Unless otherwise stated, all data in this study are presented as average ± standard deviation. Statistical analyses were performed in Origin 9.0 software. The Shapiro–Wilk normality test was applied to determine the appropriate statistical approach. For datasets following a normal distribution, a one‐way ANOVA with Tukey post‐hoc test was performed. For non‐normally distributed data, Kruskal–Wallis ANOVA with Dunn's post‐hoc test was used. Statistical significance was evaluated at a threshold of α = 0.05 for all analyses.

## Conflicts of Interest

The authors declare no conflicts of interest.

## Supporting information




**Supporting File**: mabi70131‐sup‐0001‐SuppMat.docx.

## Data Availability

The data that support the findings of this study are available from the corresponding author upon reasonable request.
