# Supplementary material for: Calcium Phosphate Mineralization of Bulk Alginate Hydrogels Composites With Metal and Metal Oxide Nanoparticles
Source: Macromol Biosci. 2026 Jan 20;26(1):e00468. doi: 10.1002/mabi.202500468 (PMC12818023; doi:10.1002/mabi.202500468)
Supplement: Supplementary file 1 — Supporting File: mabi70131‐sup‐0001‐SuppMat.docx. [file MABI-26-e00468-s001.docx]

Supporting Information

Calcium Phosphate Mineralization of Bulk Alginate Hydrogels Composites with Metal and Metal Oxide Nanoparticles

Ana-Marija Milisav, Vida Strasser, Andrea Marfoglia, Krunoslav Bojanić, Ina Erceg, Silke Christiansen, Maja Ivanić, Željka Fiket, Sophie Cazalbou, Maja Dutour Sikirić*

Contents:

S1 Characterization of nanoparticles

S2 Macroscopic cross-sectional morphology of mineralized 1 % alginate hydrogels

S3 Frequency sweep of 1 % alginate hydrogels

S1 Characterization of nanoparticles

Three types of NPs, namely AgNPs, CuONPs and ZnONPs, known for their broad antibacterial activity and low probability of inducing bacterial resistance,^[1]^ were selected for the preparation of NCHs and mineralized NCHs. Their PXRD patterns and SEM micrographs are given in Fig. S1.1, while FTIR spectra are shown on Fig. S1.2.

The PXRD pattern of AgNPs contained the peaks at 2*θ* 38.1°, 44.2° and 64.4°, which correspond to the (111), (200) and (220) reflections of silver, respectively (JCPDS, File No. 4-0783). No bands characteristic of the Ag-Ag bond vibration were observed in the FTIR spectrum (not shown) as they do not appear in the 4000–400 cm^−1^ region.^[2]^ The SEM and TEM micrograph revealed that the AgNPs have an irregular polyhedral morphology and an average particle size of 21.8 ± 13.7 nm.

In the PXRD pattern of CuONPs, peaks at 2*θ* 32.4°, 35.4°, 38.7°, 46.3°, 48.7°, 53.5°, 58.3°, 61.5°, 66.2°, and 68.1°*,* corresponding to (110), $\left( \bar{\boldsymbol{1}}\boldsymbol{11} \right)$, (111), $\left( \bar{\boldsymbol{1}}\boldsymbol{12} \right)$, $\left( \bar{\boldsymbol{2}}\boldsymbol{02} \right)$, (020), (202), $\left( \bar{\boldsymbol{1}}\boldsymbol{13} \right)$, $\left( \bar{\boldsymbol{3}}\boldsymbol{11} \right)$, (220) reflections of monoclinic CuO (JCPD No. 01-080-0076) ^[3,4]^ were observed, respectively. The FTIR spectrum (Fig. S1.2) contained the bands at 600 cm^−1^ and 480 cm^−1^, which can be assigned to the vibrations of Cu-O bond in monoclinic cupric oxide.^[5,6]^ The CuONPs had an irregular, elongated, plate-like morphology with an average size of 60.4 ± 18.7 nm.

The PXRD pattern of the ZnONPs contained peaks at 2*θ* 31.7°, 34.4°, 36.2°, 47.5°, 56.5°, 62.8°, 66.3°, 67.9°, and 69.1° corresponding to reflections (100), (002), (101), (102), (110), (103), (200), (112) and (201), respectively (JCPDS-ICDD 36-1451). The FTIR spectrum (Fig. S1.2) contained a band at 667 cm^−1^ that can be attributed to the hexagonal ZnO phase.^[7]^ The SEM and TEM micrographs revealed that the ZnONPs were plate-like and had a size of 75.0 ± 34.5 nm.


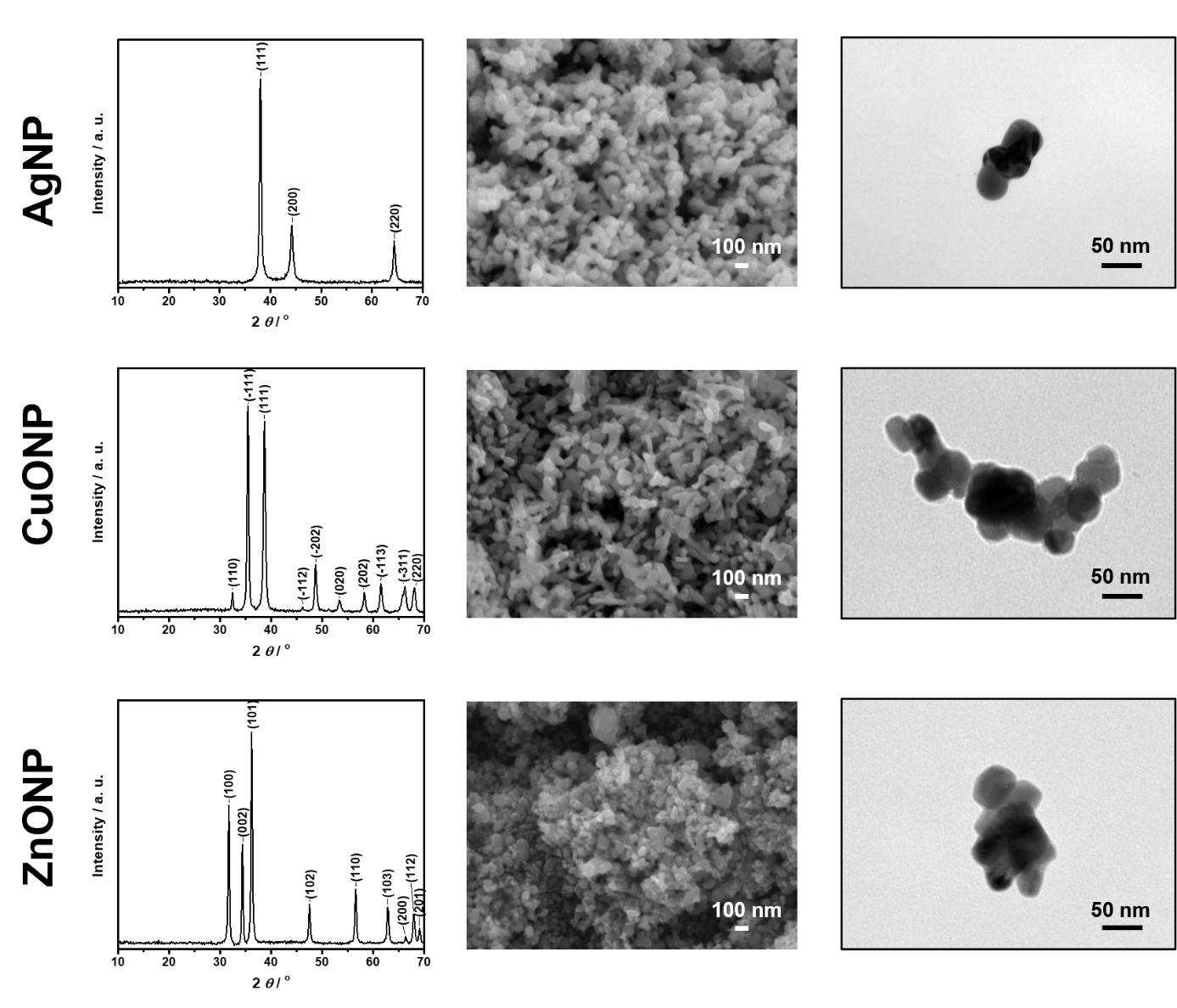


**Figure S1.1** Powder X-ray diffraction patterns and scanning electron micrographs of the silver (AgNP), copper oxide (CuONP), and zinc oxide (ZnONP) nanoparticles.





**Figure S1.2** Fourier transform infrared spectroscopy of copper oxide (CuONPs) and zinc oxide (ZnONPs) nanoparticles.

S2 Macroscopic cross-sectional morphology of mineralized 1 % alginate hydrogels


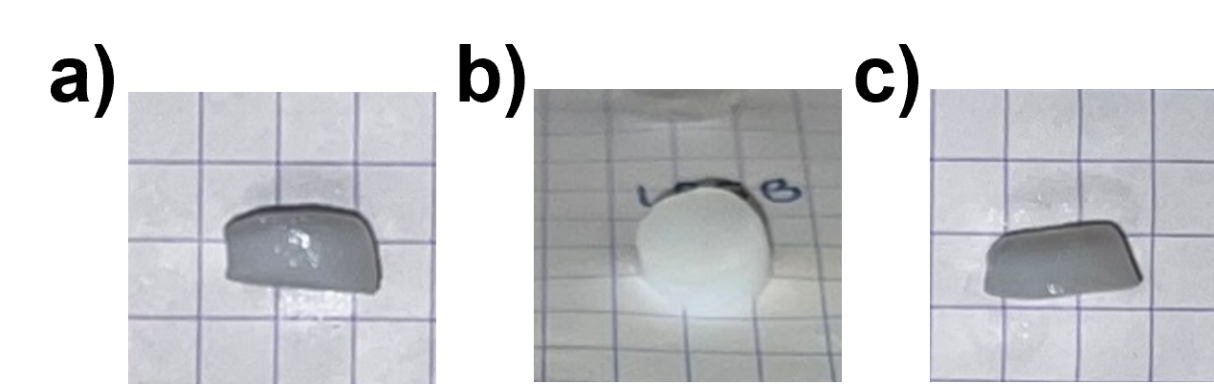


**Figure S2.1** Lateral appearance of 1 % alginate hydrogels prepared at a-b) pH 9.0, mineralized with calcium phosphates (B1) and c) one incorporating Ag nanoparticles (B1Ag) showing the distribution of the mineralized layer.

S3 Frequency sweep of 1 % alginate hydrogels


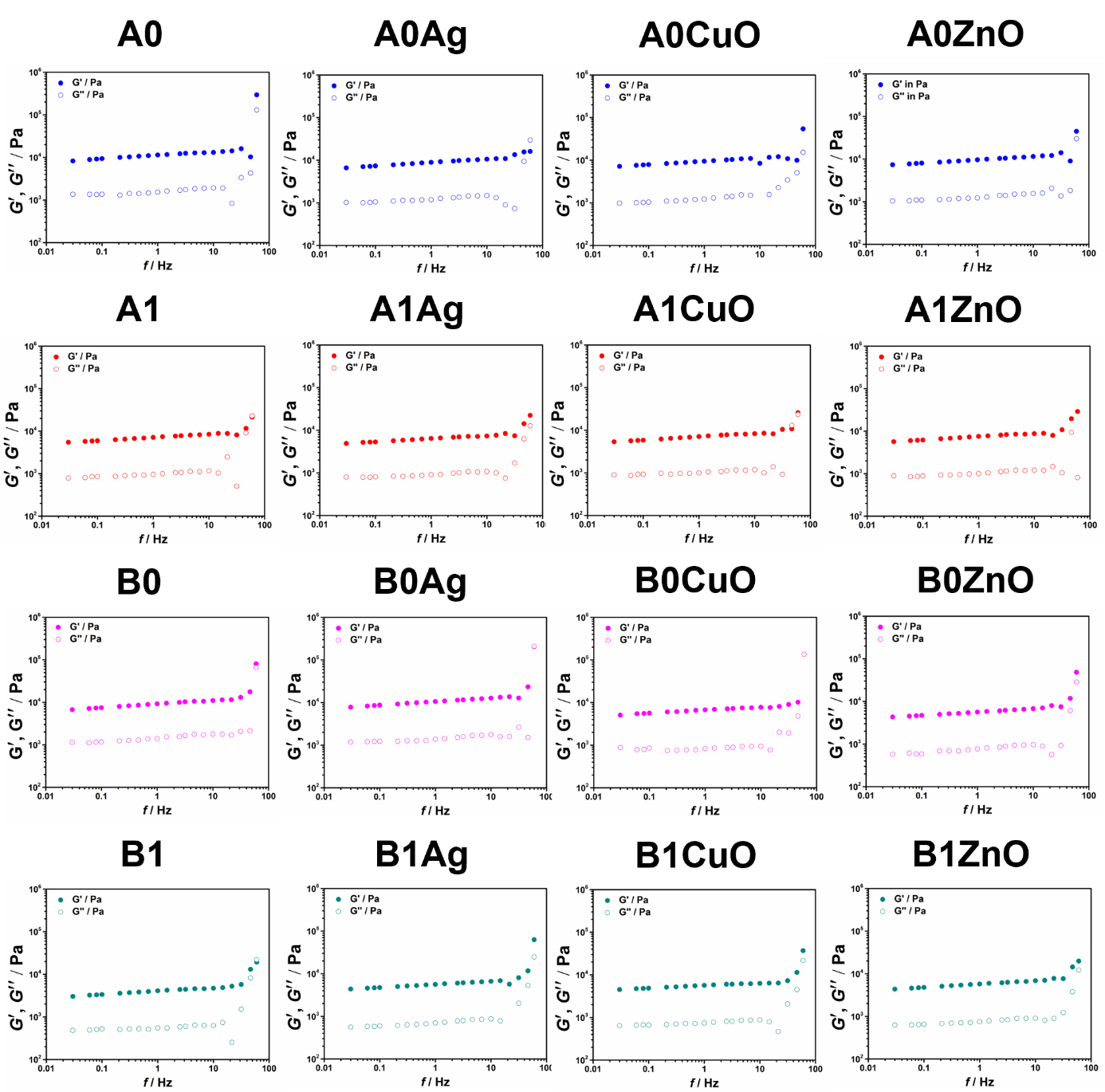


**Figure S3.1** Frequency sweep analysis of 1 % alginate hydrogels, both non-mineralized (A0, B0) and calcium phosphate mineralized (A1, B1), incorporating Ag, CuO and ZnO nanoparticles prepared in TRIS buffer at either pH 7.4 (A0, A1) or pH 9 (B0, B1), with the dependence of storage modulus (*G*') and loss modulus (*G*'') moduli over frequency. Full dots: *G*', empty dots: *G*'', 25 °C.

References:

[1] S. Sheikh-Oleslami, B. Tao, J. D’Souza, F. Butt, H. Suntharalingam, L. Rempel, N. Amiri, Gels 2023, 9, 591.

[2] L. Gharibshahi, E. Saion, E. Gharibshahi, A. Shaari, K. Matori, Materials 2017, 10, 402.

[3] D. O. B. Apriandanu, Y. Yulizar, Nano-Structures & Nano-Objects 2019, 20, 100401.

[4] S. A. Ekanayake, P. I. Godakumbura, ACS Omega 2021, 6, 26262.

[5] R. Jana, A. Dey, M. Das, J. Datta, P. Das, P. P. Ray, Applied Surface Science 2018, 452, 155.

[6] X. Liu, Z. Li, Q. Zhang, F. Li, T. Kong, Materials Letters 2012, 72, 49.

[7] W. Muhammad, N. Ullah, M. Haroon, B. H. Abbasi, RSC Adv. 2019, 9, 29541.
